# Successful Stent Graft Insertion for Endovascular Aneurysm Repair and Closure of Patent Ductus Arteriosus in an Adult Patient

**DOI:** 10.1155/2015/317061

**Published:** 2015-02-01

**Authors:** Toshiki Kuno, Koji Tsutsumi, Yohei Numasawa

**Affiliations:** ^1^Department of Cardiology, Ashikaga Red Cross Hospital, Yobechou 284-1, Ashikaga, Tochigi 326-0843, Japan; ^2^Department of Cardiovascular Surgery, Ashikaga Red Cross Hospital, Tochigi, Japan

## Abstract

Patent ductus arteriosus (PDA) is sometimes undetected until adulthood, and surgical closure of a PDA is dangerous because of the calcification of the ductus. Percutaneous approaches such as coil embolization and use of a PDA occluder are less invasive; however, these devices are not suitable for PDA with thoracic aortic aneurysm (TAA). We present the case of a 72-year-old female patient who underwent successful stent graft insertions for PDA with TAA.

## 1. Introduction

Patent ductus arteriosus (PDA) is usually diagnosed and treated during early childhood; however, it is sometimes undetected until adulthood. For older patients, surgical PDA ligation is highly dangerous because of the calcification of the ductus. Endovascular treatment is a less invasive method. Therefore, we report the case of a 72-year-old female patient who underwent successful stent graft insertions for PDA with thoracic aortic aneurysm (TAA).

## 2. Case Report

**Figure 1:**
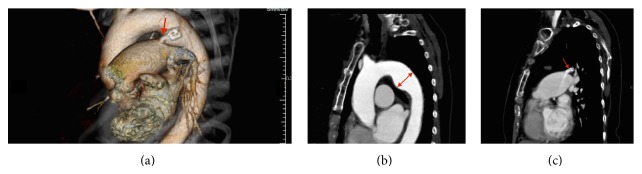
(a) A volume rendering CT image showing the PDA (red arrow). (b) A curved planar CT reformation image showing the TAA (red arrow; diameter, 41 mm). (c) A curved planar CT reformation image showing the PDA (red arrow; diameter, 6 mm) with contrast enhancement and calcification.

A 72-year-old female patient with the history of hypertension was found to have PDA on a transthoracic echocardiography in June 2009. She had no symptoms at that time. In March 2013, she complained of mild dyspnea of New York Heart Association class II. Computed tomography (CT) revealed a 41 mm TAA with calcified PDA with a diameter of 6 mm (Figures 1(a)–1(c)). In May 2013, cardiac catheterization was performed, with the following results: pulmonary-to-systemic flow ratio, 2.0; mean pulmonary artery wedged pressure, 15 mm Hg; and mean pulmonary artery pressure, 25 mm Hg. These results and the clinical manifestations indicated the need for the closure of the PDA; however, the diameter was too large for coil embolization and the Amplatzer Duct Occluder (AGA Medical, Golden Valley, MN, USA) was not suitable because of the TAA. Generally, open surgery for PDA ligation is very difficult for the elderly because cardiopulmonary bypass is required and the ductus has calcification. Therefore, we chose to treat the TAA and PDA with stent graft insertions.

**Figure 2:**
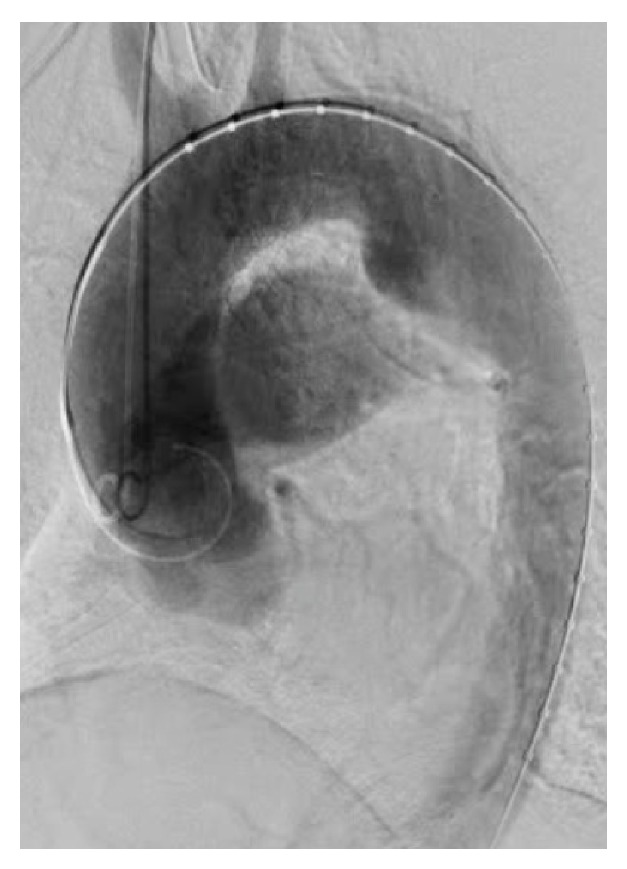
An aortography image (LAO 45) showing the connection between the TAA and the pulmonary artery.

**Figure 3:**
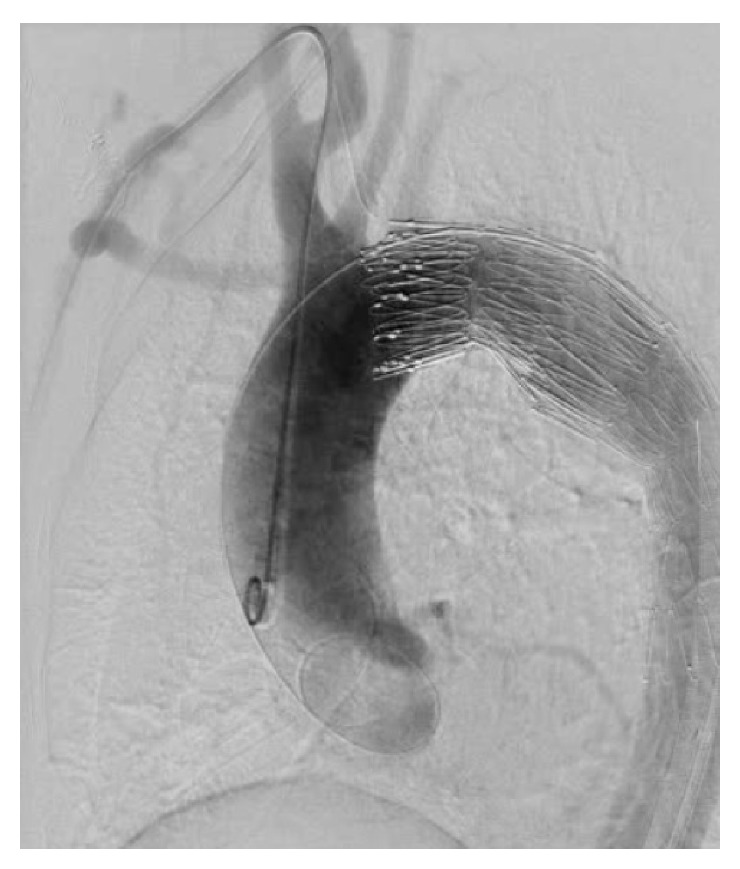
An aortography image (LAO 45) showing the disappearance of the connection between the TAA and the pulmonary artery after stent graft insertions.

**Figure 4:**
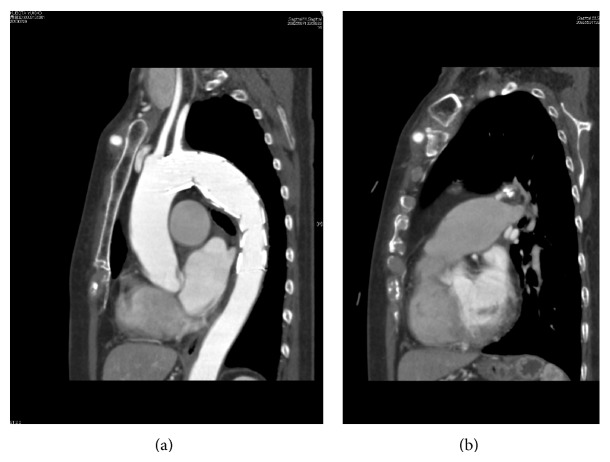
(a) A curved planar CT reformation image showing the TAA with stent grafts. (b) A curved planar reformation CT image showing the PDA without contrast enhancement. CT, computed tomography; PDA, patent ductus arteriosus; TAA, thoracic aortic aneurysm; LAO, left anterior oblique.

With the patient under general anesthesia, bypass from the right subclavian artery to the left subclavian artery was performed with a Gore Tex ring graft (W.L. Gore and Associates, Newark, DE, USA) because the landing zone between the TAA and the left subclavian artery was only 14 mm (Figures 1(a) and 1(b)) and a high risk of occlusion of that artery was suspected. Then, a 5 Fr sheath was inserted through the right brachial artery, and a pig-tailed-type catheter for aortography was placed in the ascending aorta. Aortography showed the connection between the TAA and the pulmonary artery (Figure 2 and Video 1 in Supplementary Material available online at http://dx.doi.org/10.1155/2015/317061). The stent delivery system (24 Fr sheath) was inserted through the right femoral artery with open surgery exposure. A 36 mm × 157 mm Cook Zenith TX2 stent graft (Cook Medical, Bloomington, IN, USA) was placed in the descending thoracic aorta; however, type 1 endoleak occurred and, therefore, a 36 mm × 77 mm Cook Zenith TX2 stent graft was deployed and the endoleak disappeared. Aortography revealed the occlusion of the PDA (Figure 3 and Video 2). After the procedures, transthoracic echocardiography showed no continuous flow into the pulmonary artery and CT demonstrated the inserted stent graft in the thoracic aorta and no enhancement of the PDA (Figures 4(a) and 4(b)). The patient was discharged on the seventh day, and she did not complain of dyspnea on exertion.

## 3. Discussion

PDA is usually diagnosed and treated during early childhood; however, it is sometimes undetected until adulthood. PDA with a minimal diameter of 6 mm can itself cause significant pulmonary blood overflow. In adults, treatments are aimed at preventing decompensated heart failure and endocarditis. For older patients, surgical PDA ligation is highly dangerous because of the calcification of the ductus, and surgery after age 60 years is controversial [1].

Endovascular treatment is a less invasive method. The Amplatzer Duct Occluder has high success rates (up to 12 mm diameter) [2], and coil embolization is normally done for PDAs <4 mm in diameter; however, with the use of a 0.052 in Gianturco Coil (Cook Medical), the maximum diameter was up to 5.6 mm [3]. In our case, these devices were not appropriate because of the TAA. Furthermore, because we needed to treat the TAA itself, we chose to perform treatment with the stent graft.

In cases similar to ours, stent grafts were used for PDA and PDA aneurysmal formation [4, 5]. However, this is the first case in which stent grafting was used for TAA and PDA. The landing zone for the stent graft is important because the PDA is often near the left subclavian artery. In our case, because the landing zone was not enough (Figures 1(a), 1(b), and 2), we added a bypass from the right subclavian artery to the left subclavian artery to prevent cerebral infarction. We successfully treated the PDA and TAA without complications.

## 4. Conclusion

This was the first case of stent grafting for TAA and PDA. Stent grafts may be considered if coil embolization and the Amplatzer Duct Occluder are not appropriate.

## Supplementary Material

Video 1: An aortagraphy with a 5Fr pig-tailed-type catheter through the right brachial artery. An aortagraphy showed the connection between the thoracic aortic aneurysm and the pulmonary artery.
Video 2: An aortagraphy with a 5Fr pig-tailed-type catheter through the right brachial artery. An aortagraphy revealed the occlusion of the patent ductus arteriosus.
